# Higher Polygenetic Predisposition for Asthma in Cow’s Milk Allergic Children

**DOI:** 10.3390/nu10111582

**Published:** 2018-10-27

**Authors:** Philip R. Jansen, Nicole C. M. Petrus, Andrea Venema, Danielle Posthuma, Marcel M. A. M. Mannens, Aline B. Sprikkelman, Peter Henneman

**Affiliations:** 1 Department of Complex Trait Genetics, Center for Neuroscience and Cognitive Research, Amsterdam Neuroscience, VU University, 1081 HV Amsterdam, The Netherlands; p.r.jansen@vu.nl (P.R.J.); danielle.posthuma@vu.nl (D.P.); 2 Department of Child and Adolescent Psychiatry, Erasmus MC, 3015 GD Rotterdam, The Netherlands; 3 Department of Pediatric Respiratory Medicine and Allergy, Emma Children’s Hospital, AUMC, 1105 AZ Amsterdam, The Netherlands; n.c.petrus@amc.nl (N.C.M.P.); a.b.sprikkelman@umcg.nl (A.B.S.); 4 Department Clinical Genetics, Genome Diagnostics Laboratory, AUMC, 1105 AZ Amsterdam, The Netherlands; a.venema@amc.uva.nl (A.V.); m.a.mannens@amc.nl (M.M.A.M.M.); 5 Department of Pediatric Pulmonology and Allergology, Beatrix Children’s Hospital, UMCG, 9713 GZ Groningen, The Netherlands

**Keywords:** allergic march, cow’s milk allergy, genome-wide association, polygenic risk score

## Abstract

Cow’s milk allergy (CMA) is an early-onset allergy of which the underlying genetic factors remain largely undiscovered. CMA has been found to co-occur with other allergies and immunological hypersensitivity disorders, suggesting a shared genetic etiology. We aimed to (1) investigate and (2) validate whether CMA children carry a higher genetic susceptibility for other immunological hypersensitivity disorders using polygenic risk score analysis (PRS) and prospective phenotypic data. Twenty-two CMA patients of the Dutch EuroPrevall birth cohort study and 307 reference subjects were genotyped using single nucleotide polymorphism (SNP) array. Differentially genetic susceptibility was estimated using PRS, based on multiple *P*-value thresholds for SNP inclusion of previously reported genome-wide association studies (GWAS) on asthma, autism spectrum disorder, atopic dermatitis, inflammatory bowel disease and rheumatoid arthritis. These associations were validated with prospective data outcomes during a six-year follow-up in 19 patients. We observed robust and significantly higher PRSs of asthma in CMA children compared to the reference set. Association analyses using the prospective data indicated significant higher PRSs in former CMA patients suffering from asthma and related traits. Our results suggest a shared genetic etiology between CMA and asthma and a considerable predictive sensitivity potential for subsequent onset of asthma which indicates a potential use for early clinical asthma intervention programs.

## 1. Background

Cow’s milk allergy (CMA) is among the most frequent food allergies in young children [1]. An exact incidence, however, is difficult to establish, since it has been shown that large discrepancies exist between self-reported and proper clinically diagnosed CMA [2,3,4]. As with most other allergies, CMA has a complex and heterogeneous clinical presentation. The heritability of CMA is estimated at 15%, which, when compared to other (food) allergies, represents a moderate genetic component [5,6]. Although most young children develop tolerance for cow’s milk proteins within a few years, these children seem to have an increased risk for developing other diseases involving a hypersensitive immune system, including asthma and inflammatory bowel disease (IBD) [6,7,8,9,10], which may suggest common genetic pathways between these diseases. While several genome-wide surveys on asthma, allergic rhinitis (AR) and atopic dermatitis (AD) have been reported, genome-wide association studies (GWAS) of food allergy (FA) are currently still limited [11,12,13,14]. Recently, we reported a candidate-gene association study in CMA-children and matched controls [15]. Although the results of the latter study favoured the “Allergic March” hypothesis, the number of studied variants was limited and the specific direction of the allergic march was narrowly defined [11,16]. Asthma, AD, and rheumatoid arthritis (RA) have previously been suggested to be related to CMA [17,18]. In addition to these immunological hypersensitivity related disorders, human studies also revealed a clear link between autism spectrum disorder (ASD) and asthma, and related immunological sensitivities which strongly suggest that allergic diseases are common in patients with ASD [19,20]. In 2013 Theoharides suggested that activation of brain mast cells and immune factors are associated with behavioural and language development, which may lead to focal immune reactions in the brain and subsequent focal encephalitis [21].

Large-scale GWAS studies have identified large numbers of single nucleotide polymorphisms (SNPs) related to behavioural and disease-related traits [22]. Moreover, studies have shown that the vast majority of these traits are highly polygenic, showing evidence of a complex genetic architecture composed of many genetic variants of low individual effect. However, the variability that can be explained by results from current GWAS studies is much lower than the actual heritability of these traits, commonly referred to as the ‘missing heritability’ [23]. Despite a large gap between variance explained by GWAS and the total variance of the trait, it has been shown that marginally significant SNPs that do not reach genome-wide significance (*P* < 5 × 10^−8^) contribute to the explained variance in the trait [24]. The genetic signals of these sub-threshold markers can be collectively captured by a polygenic risk score (PRS) that includes significant and non-significant markers, quantifying the genome-wide genetic predisposition of an individual for a specific trait [25,26]. In addition, such a genome-wide score analysis can also be used to study a shared genetic susceptibility across traits and diseases.

In this study, we explore the contribution of common genetic variants associated with asthma, ASD, AD, IBD, and AR, captured by PRS, to patients that have suffered from CMA by comparing the PRSs of CMA children with the PRS of healthy controls. Secondly, using six-year follow-up data from these children we aim to link the PRS for these traits to phenotypes related to asthma, AR and AD. Put together, the results of this study may provide new insight into a possible shared genetic architecture between CMA and these traits.

## 2. Methods

Sample collection: In this study, we included 22 children, suffering from CMA at intake and participating in the Dutch EuroPrevall Birth Cohort study. All 22 children became cow’s milk tolerant within a time frame of two years after diagnosis. The EuroPrevall study is described in detail in Supplementary File S1 and previously by others [4,27,28]. Follow-up data of the Dutch EuroPrevall Birth Cohort study were acquired between 2015 and 2016, when the children reached the age of approximately six years old. The follow-up study involved questionnaires regarding (food) allergy related symptoms and, when indicated, further medical examination. Prospective data were obtained for symptoms indicating (1) asthma: wheezing, dyspnoea, (nightly) coughing, clinically diagnosed asthma and asthma medication use over the last 12 months, (2) allergic rhinitis (AR): permanent irritated nasal mucosa, burning sensation of the eyes, clinically diagnosed AR and use of AR medication over the last 12 months, (3) atopic dermatitis (AD): eczema and the (cutaneous) use of topical steroids, and (4) any food allergy. The present study only focused on the questionnaire outcomes related to asthma, AR and AD and their clinical diagnosis and/or the use of anti-allergy medication. Complete follow-up data was available for 19 former CMA patients. The selection of this subset of CMA cases was done irrespective of health status, i.e., no information of the above described health status was available at the time of genotyping.

All children described in our manuscript are participating in the Dutch EuroPrevall birth cohort study. The Medical Ethics Committee of the AUMC approved the Dutch EuroPrevall Birth Cohort Study (METC 2006/005 and METC prospective data 2014/056). Written informed consent, for both the study and genetic sampling, was obtained from both parents of each child, unless only one of them had parental rights. Within the context of a reference group, we selected 307 anonymous famine unexposed controls from the “Dutch Famine cohort”. The subjects of this reference set were, like our CMA cases, born in the vicinity of Amsterdam, The Netherlands, and born in the period between 1946 and 1947, sampled over the last two decades. This reference set is assumed to represent the general Dutch population rather than screen negatives for any studied trait in this study. According previous reports on the Dutch Famine cohort, we assumed for allergies and related phenotypes, that this reference set reflects the general prevalence’s in the Dutch population [29] For the reference set, the Medical Ethics Committee of the AUMC approved the study (METC 2001/215) and a written informed consent was obtained for each participant [29,30].

Genotyping, quality control, and genotype imputation and polygenic scoring: amplified genomic (see Supplementary File S1) and genomic DNA of patients and reference set, respectively, were submitted for microarray-based genotyping at GenomeScan B.V. (Leiden, The Netherlands), using the Affymetrix Axiom UKB WCSG-96 array (Santa Clara, CA, USA). Imputation of the cleaned genotype dataset was performed using IMPUTE2 [31]. PRSs were calculated based on genome-wide association (GWAS) results for the following five traits: (1) Asthma, reported by Moffatt et al. in 2010 [32], (2) ASD, reported by the Cross-Disorder Group of the Psychiatric Genomics Consortium in 2013 [33], (3) AD, reported by Paternoster et al. in 2015 [13], (4) IBD, reported by Liu et al. in 2015 [34] and (5) RA reported by Stahl et al. in 2010 [35]. In order to evaluate the optimal *P*-value threshold (*P_T_* ) for discriminating cases and the reference set, we calculated additive PRSs on a broad range of *P*-value thresholds for SNP inclusion (*P_T_* < 0.001, *P_T_* < 0.005, *P_T_* < 0.01, *P_T_* < 0.05, *P_T_* < 0.1, *P_T_* < 0.5, and *P_T_* < 1). Polygenic scores were calculated by multiplying the number of risk alleles (0, 1, 2) by the SNP effect size (beta or log-transformed odds ratio). This study aims to detect genetic predisposition for several other adverse immunological outcomes after suffering from cow’s milk allergy per se, in relation to the general Dutch population. In this context, the age difference between cases and control was assumed not to be a limiting factor and we considered the reference set in this study to be appropriate. Prior to statistical analyses, PRS values were standardized to a mean of 0 and standard deviation of 1. In this study, we analysed common variants, implying that after this standardization the risk alleles in the reference set would be normally distributed centred around 0. In the former CMA patients, for any trait analysis, a deviation of the mean from 0 indicates enrichment of risk alleles. Detailed descriptions of genotyping, quality control, imputation, polygenic risk scoring and further statistical analyses can be found/have been included in Supplementary File S1. Genotypes of the used SNPs, including imputation score (RSQ), of all included cases and the reference set and the appropriate meta-data that were used in this study are available on reasonable request and, according to the Dutch privacy law, only after a data transfer agreement.

## 3. Results

Genotype sample: The mean call rates in CMA cases and the reference set were 97.1% and 97.7% respectively (Table 1). Although in both the cases and the reference set we observed a single sample with a call rate lower than 95%, subsequent data evaluation did not show any other deviation of standard (GenABEL) SNP array quality checks in these samples. Therefore, no samples were excluded in further analyses. Imputation to the 1000 Genomes reference panel (phase1, v3 (20101123, GRCh37 / hg19) yielded 27,507,174 genetic variants. Post-imputation quality control, based on minor allele frequency (MAF > 0.01) and INFO score (INFO > 0.9), yielded a total number of 6,932,124 variants present in all samples which were available for calculating PRS.

**Figure 1:**
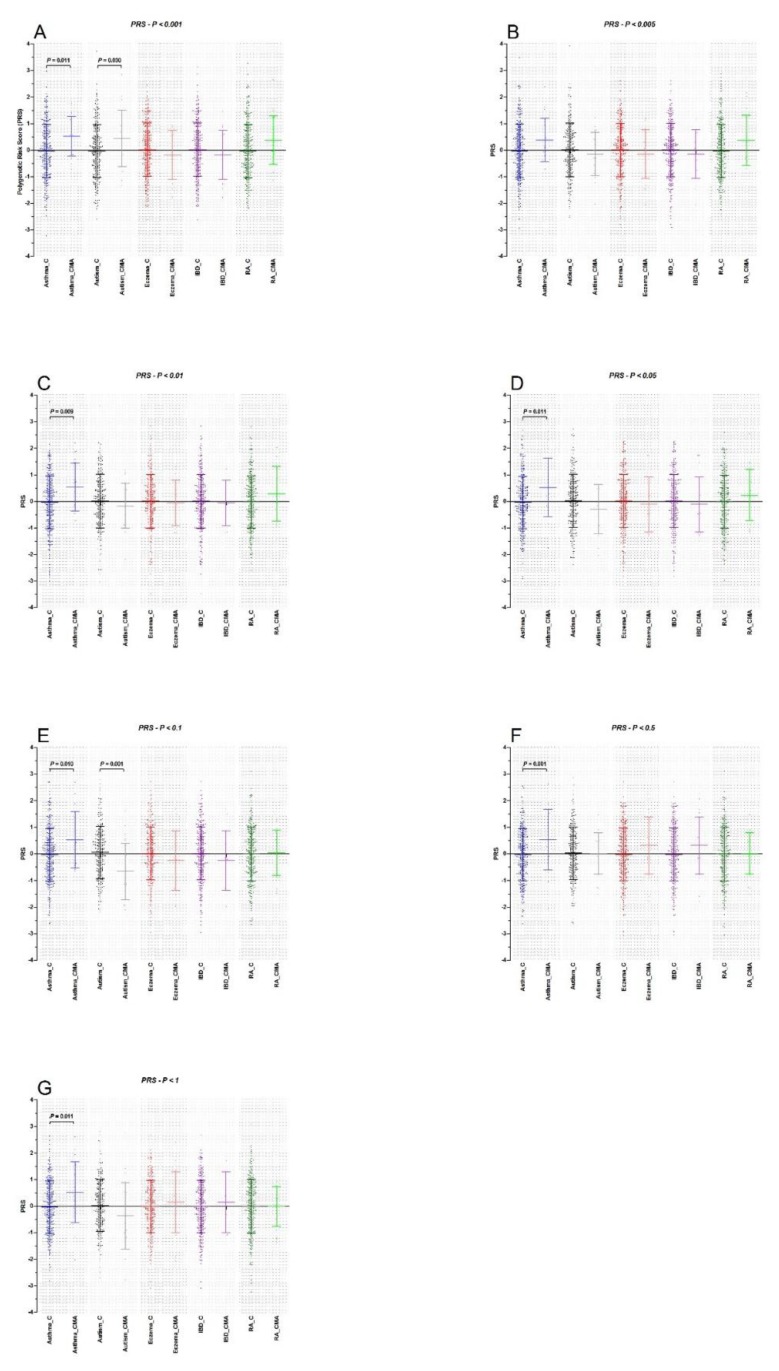
Polygenic risk scoring (weighted) for hypersensitive immune disorders asthma, autism spectrum disorder, atopic eczema, inflammatory bowel disease and allergic rhinitis (Prism 5.01, 2007). C: represents PRS in controls (*N* = 307), CMA represent PRS in (former) cow’s milk allergic children (*N* = 22). (**A**) PRS scoring with (GWAS) cutoff of *P_T_* < 0.001, (**B**) PRS scoring with (GWAS) cutoff of *P_T_* < 0.005, (**C**) PRS scoring with (GWAS) cutoff of *P_T_* < 0.01, (**D**) PRS scoring with (GWAS) cutoff of *P_T_* < 0.05, (**E**) PRS scoring with (GWAS) cutoff of *P_T_* < 0.1, (**F**) PRS scoring with (GWAS) cutoff of *P_T_* < 0.5 and (**G**) PRS scoring with (GWAS) cutoff of *P_T_* < 1. Parametric test (*t*-test) was performed to test for differences in means of the PRS between former CMA patients and healthy controls. We assumed a *P* < 0.05 statistically significant.

Polygenic risk scores (PRS): PRS analyses on imputed genotype data of CMA and the reference set were performed using SNP sets obtained for each trait. The number of SNPs that entered each PRS analysis is described in Table S1. We observed a significantly higher PRS in CMA cases compared to the reference set for all SNP set *P*-value thresholds for asthma, with the exception of the *P*-value threshold of *P_T_* < 0.005 that showed a similar direction of effect, but did not yield a significant differential association (Figure 1, Table 2). The two largest mean asthma PRS differences (δ) between cases and the reference set were observed for the *P*-value thresholds *P_T_* < 0.005 (δ = 0.41, OR = 1.50, 95% CI = 0.98–2.32, *P* = 0.065) and *P_T_* < 0.01 (δ = 0.58, OR = 1.85 95% CI = 1.16–2.94, *P* = 0.009) (Figure 1, Table 2). For the ASD PRS, we observed a significantly higher PRS for the SNP set *P_T_* < 0.001 in CMA cases compared to the reference set. However, for all other ASD SNP sets we found opposite trends in CMA cases of which the PRS SNP set *P_T_* < 0.1 was significantly lower (*P =* 0.001) in CMA patients compared to the reference set (Figure 1). Although we found no significant differences between cases and the reference set for all *P_T_* thresholds of the AD PRS analyses, our analyses showed a consistent trend to a negative PRS in CMA cases compared to the reference set. Similar findings were observed for Inflammatory bowel disease SNP sets, consistently showing lower PRS in CMA cases than in the reference set (Figure 1, Table 2), although not reaching significance. Finally, for RA we observed suggestively higher PRS in CMA patients compared to the reference set, with the exception of the largest SNP set of *P_T_* < 1 (Figure 1, Table 2).

**Figure 2:**
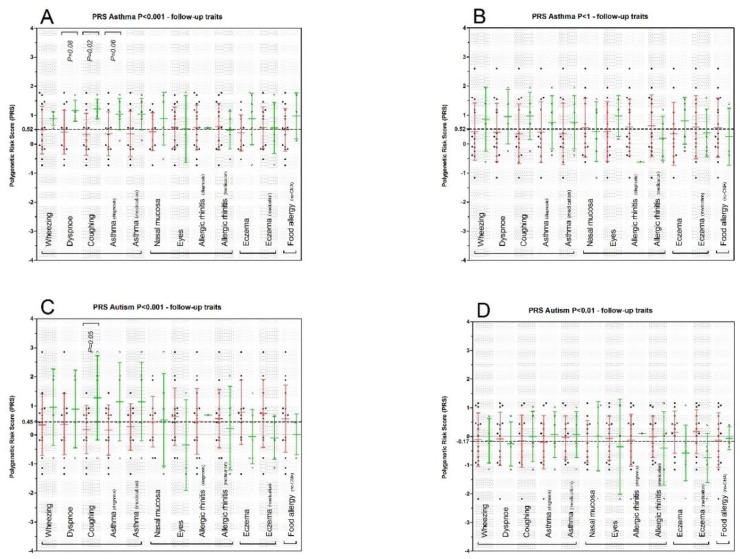
Polygenic risk scoring (weighted) for hypersensitive immune disorders asthma and autism spectrum disorder in relation to (prospectively obtained) hypersensitive immunological traits (Prism 5.01, 2007): (i) asthma related: wheezing; dyspnoe, (nightly) coughing; asthma clinically diagnosed; asthma medication (past 12 months), (ii) allergic rhinitis related: chronically irritated nasal mucosa, chronically irritated eyes, allergic rhinitis clinically diagnosed, Allergic rhinitis medication (past 12 months), (iii) atopic dermatitis: eczema; topical steroids use (past 12 months), (iiii) food allergy. (**A**) Asthma PRS scoring with (GWAS) cutoff of *P_T_* <0.001 and (**B**) asthma PRS scoring with (GWAS) cutoff of *P_T_* < 1. (**C**) ASD PRS scoring with (GWAS) cutoff of *P_T_* < 0.001 and (**D**) ASD PRS scoring with (GWAS) cutoff of *P_T_* < 0.01. ANOVA was performed to test for differences in means of the PRS between CMA patients scored for particular symptom or not. Red indicates no, green indicates yes. We assumed a *P* < 0.05 statistically significant.

Association of PRS with follow-up allergy related traits: Characteristics of the subset of CMA patients (*N* = 19, six females), obtained when they were around six years of age, are described in Table 3. In general, the highest incidence we observed concerned asthma-related and AD-related symptoms. In the latter context, we observed that diagnosed asthma, asthma medication use, AD and the use of topical steroids at the age of six years were present in one third of the former CMA patients (Table 3). Next, we performed association analysis on each symptom within the four prospective allergic disorder symptom groups, i.e., on asthma, AR, AD, and FA. Since our sample size was small, we limited the number of tests and performed these analyses only for a subset of the PRS outcomes, namely for asthma *P_T_* < 0.001 and *P_T_* < 1 and for ASD *P_T_* < 0.001 and *P_T_* < 0.01. We assumed that allergic disorders, as obtained in the prospective data, are not independent of each other, therefore, we assumed associations with a nominal *P*-value < 0.05 as significant. For the asthma PRS *P_T_* < 0.001, we observed a positive PRS associated with nightly coughing (*P =* 0.02, Figure 2A and Table S2). However, it should be noted that both dyspnoea and clinical diagnosed asthma also showed a trend of a higher PRS. For all asthma PRS scores, with exception of *P_T_* < 0.005, we have detected a significantly higher PRS in former CMA patients. In order to address the other end of the spectrum of SNP sets, we additionally analysed the asthma extreme SNP set of *P_T_* < 1. Although not significant, this association showed similar trends with regard to the average PRS scores for the significantly associated symptoms in the asthma PRS *P_T_* < 0.001. Furthermore, one of CMA patients that showed a positive IgE level within the first 2.5 years lifespan (Table 3) showed a value >1 for all the asthma PRSs, while the other IgE positive patient showed merely negative values. We observed that dyspnoea, nightly coughing and diagnosed asthma showed relatively high average PRS of 1.0, 1.0, and 0.8, respectively (Figure 2B and Table S2).

For ASD we selected *P*-value thresholds that were significant (*P_T_* < 0.001 and *P_T_* < 0.01) to test for association with prospective data. For the PRS based on *P_T_* < 0.001, we observed significant association with nightly coughing (*P* = 0.05) and a trend (*P* = 0.07) with clinical diagnosed asthma (Figure 2C and Table S3). The PRS in the former CMA cases using the threshold of *P_T_* < 0.001 showed positive associations, while in all other *P*-value thresholds we observed protective associations of the scores (negative δ). In the prospective data analyses, we found no evidence of any association of the PRS obtained from the SNP set of *P_T_* < 0.01 with any symptom of the four disorders (Figure 2D and Table S3).

## 4. Discussion

To date, literature on the genetic architecture of common variants underlying CMA is limited to candidate gene/variant approaches, and the present study is the first to describe cross-trait association between a large number of common variants and CMA. Despite the fact that genome-wide surveys are absent and, therefore, the specific genetic architecture of CMA remains unclear, the phenotypic relation with other types of allergy have been studied widely [11,15,36]. Recently, our group and others have hypothesized that food allergy susceptibility, including CMA, involves an epigenetic component as well [37,38]. The latter hypothesis was strengthened by the fact that a genetic component cannot explain the vast increase of food allergy prevalence world-wide [7]. The way food is currently processed in comparison to the pre-industrialization period has been hypothesized to involve exposure to new antigens that might underlie the recent observed increase in allergic sensitization(s) [39]. 

On the other hand, it should be noted that clinical diagnosis of food allergies has also been improved dramatically over the last decades, which may have resulted in substantial lower number of misdiagnosis and, thus, an improved estimate of its prevalence. Moreover, given the fact that nowadays food allergy has been acknowledged as a public health issue, its diagnosis has become common practice compared to 50+ years ago, which probably also contributed to the increase of food allergies prevalence [40,41]. Furthermore, without excluding an epigenetic component contributing to the expression of food allergy, the genetic component especially for CMA might very well resemble or overlap with the genetic component of other more common types of later onset allergies, and might, therefore, represent a strong predictor for later onset hypersensitive immune disorders. 

Phenotypically, this phenomenon has been very well covered by the “allergic march” hypothesis, which was recently studied extensively by Alduraywish et al. [42]. Genetic mechanisms underlying their observations were addressed in the present study, using PRS that included common variants detected in/for other types of hypersensitive immune disorders: asthma, AR and AD. Moreover, the use of prospective data on allergy and allergic symptoms in our CMA cohort can be considered as validation tool of our findings with regard to the risk of these later onset allergies. In this context, we were able to detect consistent significantly higher PRSs for asthma for virtually all SNP *P*-value thresholds compared to the reference set, but not for AR or AD. Moreover, our asthma based findings reflected relatively high odds ratios for these PRS. These findings strongly suggest that the genetic predisposition for later onset of asthma in children with a history of CMA involves a strong overlapping genetic component that is not reflected by positive IgE plasma levels within the first 2.5 years. In order to validate these findings on the asthma PRS analyses, the availability of prospective data is essential. Our former CMA patients were followed up after approximately six years. The timing of this follow-up with regard to the manifestation of asthma or asthmatic symptoms is in line with earlier reports on disease onsets covered by the allergic march hypothesis [42]. 

Although we cannot conclude that asthma and asthma-related symptoms are more common in former CMA patients (i.e., we did not include CMA screen negatives in this context), our data does indicate that the genetic load of asthma associated common variants is enriched in the former CMA patients that suffer from asthma and asthma-related phenotypes. In the context of a potential prediction of later onset asthma in CMA patients, our symptom association results, obtained with the *P*-value threshold of *P_T_* < 0.001, are substantial. All former CMA cases that were diagnosed at a later age with asthma and/or reported nightly coughing and/or dyspnoea showed, without exception, relatively high PRS scores. The delta mean PRS difference between these patients was for dyspoea (δ = 0.74), for nightly coughing (δ = 0.88) and for asthma (δ = 0.68). A considerable number of former CMA patients showed a high PRS, but showed so far not (yet) any asthmatic symptoms. Whether these patients will develop asthmatic symptoms at a later age, should be monitored in additional follow-up studies. In case such follow-ups report that these high PRS former CMA patients do develop symptoms, this would result in a strong specificity measure as well, implying that genetic profiling of CMA patients may be a powerful tool in predicting asthmatic symptoms in later life, possibly leading to preventive interventions in the future. 

Accumulating evidence shows that a hypersensitive immune system, reflected by asthma or allergies, is associated with ASD [20]. Moreover, gastrointestinal dysfunction has also been associated with autoimmunity and ASD [19]. At the same time, gastrointestinal dysfunction, reflected for example by IBD, in relation to asthma has also been previously described [43]. In order to further explore this possible shared genetic component between allergies, asthma, ASD and IBD in our study, we performed additional PRS analyses based on/using SNP sets obtained from recently published ASD and IBD GWAS [34]. Our results yielded suggestive evidence of a shared genetic component of the smallest SNP set (*P_T_* < 0.001) of ASD in former CMA patients. Surprisingly, the other ASD *P*-value threshold, especially *P_T_* < 0.01, showed an opposite effect, reflecting an ASD favorable genetic component in former CMA patients (Figure 2C,D, Table S3). Although we cannot rule out the possibility that only a (small) subset of risk alleles may indeed be shared in CMA and ASD, these conflicting observations should be interpreted with caution. Further discussion on limitations and strengths of our study are described in Supplementary File S1. 

Future studies should aim to include larger samples of CMA cases, which allow sufficient statistical power to uncover the genetic architecture of CMA. Identification of variants and genes related to CMA may aid in identifying overlapping risk loci between CMA, allergies and immune-related traits. In conclusion, to the best of our knowledge, this is the first study to investigate polygenic risk scores and cross-trait genetic liability in CMA patients, using genome-wide SNP sets from GWAS on immune-related traits. Our PRS analyses in former CMA patients show a significantly higher PRS of immune-related traits in patients compared to the reference set and, thus, a shared genetic susceptibility of CMA and asthma and asthmatic symptoms. Moreover, on the basis of the prospective data in our former CMA patients, we found a strong indication that our PRS of asthma might contribute to an accurate prediction of later onset asthma in these patients. Improving insight into the link between genetic predisposition for immune-related traits/disorders might lead to early clinical intervention in order to prevent the manifestation of asthma or limit its severity later in life.

## Figures and Tables

**Table 1:** Characteristics cow’s milk allergic children (CMA) and the reference set.

Genotype Data	*N*	Female	Male	Age	CR (mean)	CR (min)	CR (max)
CMA	22	6	16	11.8 ± 4.9 *	97.1%	93.0%	98.0%
Reference set	307	130	177	53.3 ± 0.58 **	97.7%	94.0%	99.0%

* Mean age ± Standard deviation (months) at blood drawing. ** Mean age ± standard deviation (years) at blood drawing. CR: call-rate.

**Table 2:** Odds ratios polygenic risk score (PRS) analyses.

PRS Analysis	Asthma	ASD	AD	IBD	RA
*P_T_* < 0.001	1.79 (1.14–2.84), **0.012**	1.59 (1.04–2.44), **0.032**	0.82 (0.53–1.28), 0.383	0.82 (0.53–1.28), 0.383	0.82 (0.53–1.28), 0.383
*P_T_* < 0.005	1.50 (0.98–2.32), 0.065	0.85 (0.55–1.32), 0.481	0.86 (0.56–1.32), 0.483	0.86 (0.56–1.32), 0.483	0.86 (0.56–1.32), 0.483
*P_T_* < 0.01	1.85 (1.16–2.94), **0.009**	0.83 (0.54–1.28), 0.410	0.94 (0.61–1.45), 0.780	0.94 (0.61–145), 0.780	0.94 (0.61–1.45), 0.780
*P_T_* < 0.05	1.73 (1.13–2.27), 0.012	0.73 (0.47–1.13), 0.161	0.89 (0.58–1.37), 0.596	0.89 (0.58–1.37), 0.596	0.89 (0.58–1.37), 0.596
*P_T_* < 0.1	1.74 (1.13–2.66), **0.012**	0.45 (0.28–0.74), **0.002**	0.77 (0.50–1.18), 0.231	0.77 (0.50–1.18), 0.231	0.77 (0.50–1.18), 0.231
*P_T_* < 0.5	1.74 (1.13–2.66), **0.011**	0.67 (0.43–1.05), 0.078	1.42 (0.91–2.23), 0.124	1.42 (0.91–2.23 ), 0.124	1.42 (0.91–2.23), 0.124
*P_T_* < 1	1.73 (1.13–2.65), **0.012**	0.67 (0.43–1.04), 0.074	1.17 (0.76–1.81), 0.479	1.17 (0.76–1.81), 0.479	1.17 (0.76–1.81), 0.479

Odds ratio and the corresponding 95% confidence interval (95% CI) and corresponding *P*-value for all PRS analyses (logistic regression) based on standardized PRS values representing the effect for one standard deviation change in PRS. ASD: autism spectrum disorder; AD: atopic dermatitis; IBD: inflammatory bowel disease; RA: rheumatoid arthritis. *P*-values < 0.05 were assumed significant, annotated in bold.

**Table 3:** Six year follow-up characteristics former CMA patients.

General	
Total (*N*)	19
Female (*N*)	6
Male (*N*)	13
Age (mean ± SD)	7.0 ± 1.0 *
2 year IgE positive **	2
Asthma related	*N* (%)
Wheezing	4 (21.1%)
Dyspnoea	4 (21.1%)
Coughing at night	5 (26.3%)
Asthma diagnosed	6 (33.3%)
Asthma medication	6 (33.3%)
Allergic rhinitis related	*N* (%)
Irritated nasal mucosa)	6 (33.3%)
Eyes	3 (15.8%)
Allergic rhinitis diagnosed	1 (5.3%)
Allergic rhinitis medication	5 (26.3%)
Atopic dermatitis related	*N* (%)
Eczema	7 (36.8%)
Topical steroids	6 (31.6%)
Allergy related	*N* (%)
Food allergy	3 (15.8%)

* Mean age ± Standard deviation (years) at the time of the follow study. ** Number of CMA patients that became IgE positive (IgE < 0.35 kU/L) during the first 2.5 years of life, data on IgE levels was available for 16 of 19 former CMA patients.

## Supplementary Materials

The following are available online at http://www.mdpi.com/2072-6643/10/11/1582/s1, Supplementary File S1: supplementary methods and supplementary discussion, Table S1. Number of included SNPs (MAF > 0.01) in polygenic risk score (PRS) analyses, Table S2: Association analyses of asthma *P* < 0.001 and *P* < 1 polygenic risk score (PRS) per follow-up symptom outcome, Table S3: Association analyses of autism *P* < 0.001 and *P* < 0.1 polygenic risk score (PRS) per follow-up symptom outcome.
