# Supplementary material for: Higher Polygenetic Predisposition for Asthma in Cow’s Milk Allergic Children
Source: Nutrients. 2018 Oct 27;10(11):1582. doi: 10.3390/nu10111582 (PMC6266812; doi:10.3390/nu10111582)
Supplement: Supplementary file 1 [file nutrients-10-01582-s001.pdf]

Supplementary Material

Higher Genetic Predisposition for Asthma in Cow´s Milk Allergic Children

Supplementary file 1

Supplementary methods

Sample collection: Children were included around birth and standardized measurements were performed by questionnaires. All children with symptoms suggestive of CMA underwent, among others, a double blind placebo controlled food challenge (DBPCFC). In all CMA patients undergoing blood drawing, an attempt was made to obtain one full blood sample (EDTA KE 2.6 mL Monovette, Sarstedt BV, Etten-Leur, Netherlands) for DNA-isolation. All CMA patients became cow's milk tolerant within a time frame of two years after diagnosis. No additional blood was drawn for genetic analyses during this follow-up.

Sample preparation: DNA used in this study was extracted form peripheral blood, using an automated system for DNA extraction, Autopure LS, Qiagen®, and Gentra reagents (precipitation based), conform the manufacturer's protocol. For most children the amount of blood (<2.6 mL) and, thus, the amount of available DNA was limited. In order to obtain sufficient DNA for genome wide genotyping, we applied whole genome amplification (WGA) on 20 ng of DNA of all CMA patients using the REPLI-g mini-kit of Qiagen® conform to the manufacturer's protocol, yielding approximately 1–5 μg of amplified genomic DNA for each patient. For the (adult) reference set sufficient DNA was available.

Genotyping, quality control and genotype imputation, and polygenic scoring: Amplified genomic and genomic DNA of patients and controls respectively were submitted for microarray-based genotyping at GenomeScan B.V. (Leiden, The Netherlands), using the Affymetrix Axiom UKB WCSG-96 array (Santa Clara, USA). Each array enables genotyping of > 800K SNPs and included commonly used GWAS variants as well as exonic (coding) variants. Description about calling procedures was as follows: Array images were scanned on the GeneTitan system and genotypes were subsequently evaluated and called using the Affymetrix Power Tools (version 1.16.1). All samples in this study, showed a DQC > 0.82 and were assumed as successful. STEP 1 and STEP 2 analysis files for quality control analyses and genotyping calling (Axiom GT1 clustering algorithm) were provided by GenomeScan Leiden B.V. Annotation descriptives of all SNPs (Axiom_UKB_WCSG (version na34) was obtained from GenomeScan Leiden B.V.,originally provided by the manufacturer, Affymetrix, (Santa Clara, USA). Further Quality control steps were performed using GenABEL package (version 1.8-0, http://www.genabel.org/sites/default/files/pdfs/GenABEL-tutorial.pdf) conform to the default tutorial description, section 5.2 (cycles) of data cleaning [1]. Imputation of the cleaned genotype dataset was performed using IMPUTE2 using the 1000 Genomes reference set, phase 1, v3 (20101123) [2,3]. Imputed genotype dosages were converted to hard-call genotypes using PLINK (v1.9) [4]. Post-imputation quality control procedures consisted of filtering variants on INFO score (INFO < 0.9) and minor allele frequency (MAF < 0.01). Polygenic risk scores (PRS) were calculated based on GWAS results for the following five traits: (1) Asthma, reported by Moffatt et al. in 2010 [5]; (2) ASD, reported by the *Cross-Disorder Group of the Psychiatric Genomics Consortium* in 2013 [6]; (3) AD, reported by Paternoster *et al* in 2015 [7]; (4) IBD, reported by Liu et al. in 2015 [8]; and (5) RA reported by Stahl et al. in 2010 [9]. For all these reports, genetic variants were clumped in PLINK to obtain the most significant variant for each linkage disequilibrium (LD block (PLINK clumping parameters: window: 250 kb, $r^2 < 0.1$). Additive scores were obtained by multiplying the number of risk alleles (0,1,2) with the beta- or log-transformed odds ratio for each variant. *P*-value thresholds (P_t) for including SNPs in the PRS were set to vary between P_T< 001 and $P_T < 1$. The obtained PRS were standardized to have a mean of 0 and standard deviation of 1 to increase interpretability of the score.

Statistical analyses: In this study we evaluate genetic loads previously associated with known CMA comorbid diseases in a sample of former CMA cases and in a reference set. In these evaluations we assumed that inclusion of any of these comorbid diseases as covariate in our models would interfere with the factor of interest (genetic load per se) and thus redundant. Therefore, in none of our evaluations described below, were covariates included in the model. For each standardized PRS value (according the *p*-value threshold, P_T), a parametric test (*t*-test) was performed to test for differences in the mean PRS between cases (former CMA patients) and the reference set. Significance levels of $\alpha \leq 0.05$ were assumed statistically significant. Binary logistic regression was performed to obtain odd ratios for all PRS P_T thresholds. Odds ratios were presented as OR and the corresponding 95% confidence interval (C.I.). Association with prospective data of allergic traits with PRS were analyzed using an ANOVA test. Statistical analyses were performed in SPSS (IBM, v.24.0.0, Chicago, IL, USA).

Supplementary Discussion

Limitations and strengths of the study: A limitation of our study is the relative small sample size of our CMA cohort [10,11]. In the present study we assumed nominal *P*-values as significant. One can argue that ignoring a multiple test penalty is inappropriate. However, our considerations of not applying such a penalty was based on the fact that most tests we performed were more or less not independent of each other, the latter is per the definition required for such adjustments. These dependencies are, though the exact shared genetic architecture is unknown, supported by accumulating evidence that showed that many disorders involving a hypersensitive immune system do share important immunological proteins or pathways [12–15]. To this end, we considered our PRS tests, that were confirmed by follow-up data, as reliable and valid. Strict adjustment for multiple tests for these analyses would have resulted in too many false negative findings and misinterpretation of the true biological mechanism. However, as stated before, the results for which we were unable to validate by follow-up data, should be taken with caution. Another limiting factor was the fact that we were limited in the availability of sufficient DNA for genome-wide genotyping of the former 22 CMA patients, but not for the 307 subjects of the reference set. To overcome this issue of DNA availability, we performed genome-wide amplification only on the DNA of the former CMA patients. Although we found no evidence of considerable bias, e.g., disturbed genotypic heterozygosity or substantial numbers of deviations of the Hardy-Weinberg equilibrium, it is known that genome-wide amplification might induced some technical bias with respect to a limited number of SNPs. The variants that were affected were removed from analyses; therefore the risk that the genome-wide amplification per se affected our results was limited. Although both the reference set and former CMA patients were sampled in the Netherlands and thus the risk of population stratification is limited, we cannot exclude it. To our opinion the latter limitation was covered by the fact that we were able to validate our PRS studies on Asthma, AD and AR, using the available prospective data. For the others, i.e., ASD and IBD, we had no specific prospective information available and, thus, the prospective data was of lower meaningful value for these traits and results on these traits should be taken with a reasonable caution.

Supplementary Tables.

Table S1. Number of included SNPs (MAF > 0.01) in Polygenic Risk Score (PRS) analyses.

PRS analysis	AST	ASD	AD	IBD	RA
P < 0.001 (N)	411	487	1307	2234	722
P < 0.005 (N)	1570	1837	4696	6297	2152
P < 0.01 (N)	2721	3243	8366	10261	3699
P < 0.05 (N)	10187	11953	29733	32890	13807
P < 0.1 (N)	17429	20435	50492	54040	23727
P < 0.5 (N)	52562	64061	152621	156408	72673
P < 1 (N)	73823	90578	212966	217304	100874

PRS analyses based of clumped SNP set obtained according *P* value cut off. AST: Asthma; ASD: Autism Spectrum Disorder; AD: Atopic Dermatitis; IBD: Inflammatory Bowel Disease; RA: Rheumatoid Arthritis.

Table S2. Association analyses of asthma *P* < 0.001 and *P* < 1 Polygenic Risk Score (PRS) per follow-up symptom outcome.

**Asthma related**	**PRS AST *P* < 0.001.** symptom _ (mean ± SD)	**PRS AST *P* < 0.001.** symptom – (mean ± SD) ***P*-value***	**PRS AST *P* < 1.** (symptom - mean ± SD)	**PRS AST *P* < 1.** (symptom - mean ± SD, ***P*-value***)
Wheezing	N _ (0.5 ± 0.8)	Y _ (0.9 ± 0.2) 0.37	N _ (0.4 ± 1.0)	Y _ (0.9 ± 1.1) 0.47
Dyspnoea	N _ (0.4 ± 0.8)	Y _ (1.2 ± 0.4) 0.08	N _ (0.4 ± 1.0)	Y _ (1.0 ± 0.9) 0.35
Coughing at night	N _ (0.3 ± 0.7)	Y _ (1.2 ± 0.3) 0.02	N _ (0.4 ± 1.0)	Y _ (1.0 ± 0.8) 0.24
Asthma diagnosed	N _ (0.4 ± 0.7)	Y _ (1.0 ± 0.5) 0.06	N _ (0.4 ± 1.0)	Y _ (0.8 ± 0.9) 0.51
Asthma medication	N _ (0.4 ± 0.8)	Y _ (1.0 ± 0.5) 0.08	N _ (0.4 ± 1.0)	Y _ (0.8 ± 0.9) 0.45
**Allergic rhinitis related ***				
Irritated nasal mucosa	N _ (0.4 ± 0.7)	Y _ (0.9 ± 0.9) 0.23	N _ (0.6 ± 1.0)	Y _ (0.4 ± 1.0) 0.81
Eyes	N _ (0.6 ± 0.7)	Y _ (0.5 ± 1.2) 0.91	N _ (0.4 ± 1.0)	Y _ (1.0 ± 0.7) 0.41
Allergic rhinitis diagnosed	N _ (0.6 ± 0.8)	Y _ (0.6 ± NA) 0.98	N _ (0.6 ± 1.0)	Y _ (-0.6 ± NA) 0.25
Allergic rhinitis medication	N _ (0.6 ± 0.8)	Y _ (0.5 ± 0.7) 0.77	N _ (0.6 ± 1.1)	Y _ (0.2 ± 0.8) 0.43
**Atopic dermatitis related ***				
Eczema	N _ (0.4 ± 0.6)	Y _ (0.9 ± 0.9) 0.19	N _ (0.4 ± 1.1)	Y _ (0.8 ± 0.8) 0.36
Topical steroids	N _ (0.6 ± 0.7)	Y _ (0.6 ± 0.9) 0.97	N _ (0.6 ± 1.1)	Y _ (0.4 ± 0.8) 0.70
**Allergy related ***				
Food allergy	N _ (0.5 ± 0.8)	Y _ (1.0 ± 0.8) 0.55	N _ (0.6 ± 1.1)	Y _ (0.3 ± 1.0) 0.89

Y: Scoring on symptom= yes, N: Scoring on symptom= no. AST: Asthma; ASD Autism Spectrum Disorder. *ANOVA was performed to test for differences in means of the polygenic score between CMA patients scored for particular symptom or not. *P* < 0.05 was assumed statistically significant.

Table S3. Association analyses of autism *P* < 0.001 and *P* < 0.1 Polygenic Risk Score (PRS) per follow-up symptom outcome.

**Asthma related**	**PRS ASD *P* < 0.001.** symptom _ (mean ± SD)	**PRS ASD *P* < 0.001.** symptom – (mean ± SD) ***P*-value** *	**PRS ASD *P* < 0.1.** (symptom - mean ± SD)	**PRS ASD P < 0.1.** (symptom - mean ± SD, ***P*-value** *)
Wheezing	N _ (0.3 ± 1.0)	Y _ (1.0 ± 1.3) 0.34	N _ (-0.1 ± 0.9)	Y _ (-0.1 ± 0.8) 0.95
Dyspnoea	N _ (0.4 ± 1.0)	Y _ (0.9 ± 1.3) 0.41	N _ (-0.1 ± 0.9)	Y _ (-0.3 ± 0.8) 0.73
Coughing at night	N _ (0.2 ± 0.8)	Y _ (1.3 ± 1.5) 0.05	N _ (-0.2 ± 0.9)	Y _ (0.0 ± 0.9) 0.74
Asthma diagnosed	N _ (0.2 ± 0.9)	Y _ (1.1 ± 1.4) 0.07	N _ (-0.2 ± 0.9)	Y _ (0.1 ± 0.8) 0.55
Asthma medication	N _ (0.3 ± 1.1)	Y _ (1.1 ± 1.4) 0.11	N _ (0.0 ± 0.8)	Y _ (0.1 ± 0.8) 0.79
**Allergic rhinitis related** *				
Irritated nasal mucosa	N _ (0.5 ± 0.9)	Y _ (0.5 ± 1.6) 0.91	N _ (-0.2 ± 0.7)	Y _ (0.0 ± 1.2) 0.68
Eyes	N _ (0.6 ± 1.0)	Y _ (-0.4 ± 1.6) 0.17	N _ (-0.1 ± 0.8)	Y _ (-0.4 ± 1.7) 0.62
Allergic rhinitis diagnosed	N _ (0.5 ± 1.1)	Y _ (0.7 ± NA) 0.85	N _ (-0.1 ± 0.9)	Y _ (0.1 ± NA) 0.81
Allergic rhinitis medication	N _ (0.6 ± 1.0)	Y _ (0.2 ± 1.4) 0.58	N _ (0.0 ± 0.7)	Y _ (-0.4 ± 1.3) 0.40
**Atopic dermatitis related** *				
Eczema	N _ (0.8 ± 1.1)	Y _ (-0.1 ± 0.9) 0.11	N _ (0.2 ± 0.7)	Y _ (-0.6 ± 1.0) 0.08
Topical steroids	N _ (0.7 ± 1.2)	Y _ (-0.1 ± 0.7) 0.13	N _ (0.2 ± 0.8)	Y _ (-0.8 ± 0.9) 0.03
**Allergy related** *				
Food allergy	N _ (0.5 ± 1.1)	Y _ (0.0 ± 0.7) 0.30	N _ (-0.1 ± 1.0)	Y _ (-0.1 ± 0.4) 0.51

Y: Scoring on symptom= yes, N: Scoring on symptom= no. * ANOVA was performed to test for differences in means of the polygenic score between CMA patients scored for particular symptom or not. *P* < 0.05 was assumed statistically significant.

References

1. Aulchenko, Y.S.; Ripke, S.; Isaacs, A.; van Duijn, C.M. Genabel: An r library for genome-wide association analysis. *Bioinformatics* **2007**, *23*, 1294–1296.
2. Marchini, J.; Howie, B. Genotype imputation for genome-wide association studies. *Nat. Rev. Genet.* **2010**, *11*, 499–511.
3. Howie, B.; Marchini, J.; Stephens, M. Genotype imputation with thousands of genomes. *G3 (Bethesda)* **2011**, *1*, 457–470.
4. Purcell, S.; Neale, B.; Todd-Brown, K.; Thomas, L.; Ferreira, M.A.; Bender, D.; Maller, J.; Sklar, P.; de Bakker, P.I.; Daly, M.J., et al. Plink: A tool set for whole-genome association and population-based linkage analyses. *Am. J. Hum. Genet.* **2007**, *81*, 559–575.
5. Moffatt, M.F.; Gut, I.G.; Demenais, F.; Strachan, D.P.; Bouzigon, E.; Heath, S.; von Mutius, E.; Farrall, M.; Lathrop, M.; Cookson, W.O., et al. A large-scale, consortium-based genomewide association study of asthma. *N. Engl. J. Med.* **2010**, *363*, 1211–1221.
6. Cross-Disorder Group of the Psychiatric Genomics, C. Identification of risk loci with shared effects on five major psychiatric disorders: A genome-wide analysis. *Lancet* **2013**, *381*, 1371–1379.
7. Paternoster, L.; Standl, M.; Waage, J.; Baurecht, H.; Hotze, M.; Strachan, D.P.; Curtin, J.A.; Bonnelykke, K.; Tian, C.; Takahashi, A., et al. Multi-ancestry genome-wide association study of 21,000 cases and 95,000 controls identifies new risk loci for atopic dermatitis. *Nat. Genet.* **2015**, *47*, 1449–1456.
8. Liu, J.Z.; van Sommeren, S.; Huang, H.; Ng, S.C.; Alberts, R.; Takahashi, A.; Ripke, S.; Lee, J.C.; Jostins, L.; Shah, T., et al. Association analyses identify 38 susceptibility loci for inflammatory bowel disease and highlight shared genetic risk across populations. *Nat. Genet.* **2015**, *47*, 979–986.
9. Stahl, E.A.; Raychaudhuri, S.; Remmers, E.F.; Xie, G.; Eyre, S.; Thomson, B.P.; Li, Y.; Kurreeman, F.A.; Zhernakova, A.; Hinks, A., et al. Genome-wide association study meta-analysis identifies seven new rheumatoid arthritis risk loci. *Nat. Genet.* **2010**, *42*, 508–514.
10. Dudbridge, F. Power and predictive accuracy of polygenic risk scores. *PLoS Genet.* **2013**, 9, e1003348.
11. Spiliopoulou, A.; Nagy, R.; Bermingham, M.L.; Huffman, J.E.; Hayward, C.; Vitart, V.; Rudan, I.; Campbell, H.; Wright, A.F.; Wilson, J.F., et al. Genomic prediction of complex human traits: Relatedness, trait architecture and predictive meta-models. *Hum. Mol. Genet.* **2015**, *24*, 4167–4182.
12. Bonnelykke, K.; Sparks, R.; Waage, J.; Milner, J.D. Genetics of allergy and allergic sensitization: Common variants, rare mutations. *Curr. Opin. Immunol.* **2015**, *36*, 115–126.
13. Bonnelykke, K.; Matheson, M.C.; Pers, T.H.; Granell, R.; Strachan, D.P.; Alves, A.C.; Linneberg, A.; Curtin, J.A.; Warrington, N.M.; Standl, M., et al. Meta-analysis of genome-wide association studies identifies ten loci influencing allergic sensitization. *Nat. Genet.* **2013**, *45*, 902–906.
14. Alduraywish, S.A.; Lodge, C.J.; Campbell, B.; Allen, K.J.; Erbas, B.; Lowe, A.J.; Dharmage, S.C. The march from early life food sensitization to allergic disease: A systematic review and meta-analyses of birth cohort studies. *Allergy* **2016**, *71*, 77–89.
15. Alduraywish, S.A.; Standl, M.; Lodge, C.J.; Abramson, M.J.; Allen, K.J.; Erbas, B.; von Berg, A.; Heinrich, J.; Lowe, A.J.; Dharmage, S.C. Is there a march from early food sensitization to later childhood allergic airway disease? Results from two prospective birth cohort studies. *Pediatr. Allergy Immunol.* **2017**, *28*, 30–37.

© 2018 by the authors. Licensee MDPI, Basel, Switzerland. This article is an open access article distributed under the terms and conditions of the Creative Commons Attribution (CC BY) license (http://creativecommons.org/licenses/by/4.0/).
